# A Conserved Stem-Loop Structure within ORF5 Is a Frequent Recombination Hotspot for Porcine Reproductive and Respiratory Syndrome Virus 1 (PRRSV-1) with a Particular Modified Live Virus (MLV) Strain

**DOI:** 10.3390/v15010258

**Published:** 2023-01-16

**Authors:** Marlene Mötz, Julia Stadler, Heinrich Kreutzmann, Andrea Ladinig, Benjamin Lamp, Angelika Auer, Christiane Riedel, Till Rümenapf

**Affiliations:** 1 Institute of Virology, Department of Pathobiology, University of Veterinary Medicine Vienna, Veterinaerplatz 1, 1210 Vienna, Austria; 2 Clinic for Swine, Center for Clinical Veterinary Medicine, Faculty of Veterinary Medicine, Ludwig-Maximilians-University Munich, Sonnenstrasse 16, 85764 Oberschleissenheim, Germany; 3 Clinic for Swine, Department for Farm Animals and Veterinary Public Health, University of Veterinary Medicine Vienna, Veterinaerplatz 1, 1210 Vienna, Austria; 4 Institute of Virology, Department of Veterinary Medicine, Justus-Liebig-University Giessen, Schubertstraße 81, 35392 Giessen, Germany; 5 Département de Biologie, École Nationale Supérieure de Lyon, 46 Allée d’Italie, 69364 Lyon, France; 6 Centre International de Recherche en Infectiologie (CIRI), 46 Allée d’Italie, 69364 Lyon, France

**Keywords:** porcine reproductive and respiratory syndrome virus, PRRSV, Arteriviridae, recombination, recombinant virus, modified live virus vaccine, ORF5, stem loop

## Abstract

The emergence of recombinant PRRSV strains has been observed for more than a decade. These recombinant viruses are characterized by a genome that contains genetic material from at least two different parental strains. Due to the advanced sequencing techniques and a growing number of data bank entries, the role of PRRSV recombinants has become increasingly important since they are sometimes associated with clinical outbreaks. Chimeric viruses observed more recently are products of PRRSV wild-type and vaccine strains. Here, we report on three PRRSV-1 isolates from geographically distant farms with differing clinical manifestations. A sequencing and recombination analysis revealed that these strains are crossovers between different wild-type strains and the same modified live virus vaccine strain. Interestingly, the recombination breakpoint of all analyzed isolates appears at the beginning of open reading frame 5 (ORF5). RNA structure predictions indicate a conserved stem loop in close proximity to the recombination hotspot, which is a plausible cause of a polymerase template switch during RNA replication. Further research into the mechanisms of the stem loop is needed to help understand the PRRSV recombination process and the role of MLVs as parental strains.

## 1. Introduction

Porcine reproductive and respiratory syndrome virus (PRRSV) is an enveloped, single-stranded, positive sense RNA virus within the family *Arteriviridae*, order *Nidovirales*, genus *Betaarterivirus*, which is further divided into the subgenera *Eurpobartevirus* and *Ampobartevirus*. Each subgenus holds one species, namely *Betaarterivirus suid 1*, or PRRSV-1, and *Betaarterivirus suid 2*, or PRRSV-2. These two species are of striking genetic divergence, indicating that they might have evolved separately [1]. Furthermore, nucleotide sequences of different strains within a species show high variations caused by high mutation rates [2], missing proofreading activity of the RNA-dependent RNA polymerase (RdRp) [3], and frequent recombination of different virus strains [4]. The PRRSV genome harbors at least ten open reading frames (ORFs), of which ORF1 codes for non-structural proteins and ORFs 2–7 code for the structural proteins of the viral envelope, membrane, and nucleocapsid [5]. Clinical signs of infected animals include respiratory disorders, reproductive symptoms in sows, and the birth of weak, congenitally infected piglets [6]. A PRRSV introduction into a farm frequently causes high financial losses and makes it one of the most relevant porcine pathogens worldwide [7]. Modified live virus (MLV) vaccines are so far the only effective, commercially available, and widely used practical option to successfully combat PRRSV. However, they bear the risks of reverting to virulence [8] and recombining with wild-type strains [9,10,11]. Recombination is a common phenomenon in viruses, which requires the co-infection of one cell with two or more strains. The mechanisms of recombination differ according to the structure of the viral genome. DNA viruses tend to recombine after a double strand breaks and repairs [12] as observed in eukaryotes during homologous recombination. Segmented RNA viruses can reassort their genes after infecting the same cell as seen in *Orthomyxoviridae* [13]. Non-segmented RNA viruses, however, use a different strategy, called copy-choice replication. This phenomenon occurs during RNA replication, as the viral RdRp switches templates, resulting in a chimeric genome of two or more progenitors [14]. The same mechanism is found in retroviruses during reverse transcription [15]. Although recombination events have been associated with increased virulence [16], immune evasion, or drug resistance [15], it remains unclear whether these incidences are random or follow a strategy to modify the virus’ fitness. 

To date, several PRRSV recombinants have been isolated and characterized. These chimeric viruses are the result of the recombination of either different wild-type strains [17,18], wild-type and vaccine strains [9,10,11], or even two divergent vaccine strains [19]. Thus, the administration of PRRSV MLV vaccines, especially during an acute infection event, bears the potential of recombination with wild-type strains, and their subsequent distribution and establishment in the field. Subsequently, active characterization of circulating virus strains is an important, but not established, surveillance method for PRRSV containment.

Here we report on three PRRSV-1 isolates from Austria and Germany originating from the recombination of a wild-type strain and a specific MLV vaccine strain. Interestingly, although the strains show the highest homology to three different wild-type strains from ORF1–4, the genomic breakpoint occurs at the same location within ORF5. Hereby, we present the clinical outcome, phylogenetics, and possible recombination mechanism of these novel isolates. We hypothesize that the wild-type strains recombine with the Ingelvac PRRSFLEX^®^ EU (Boehringer Ingelheim Vetmedica GmbH, Rohrdorf, Germany)vaccine strain within ORF5. Furthermore, we propose a conserved stem-loop structure after ORF5a to be the driving force of recombination. Finally, we would like to see more detailed research into the mechanism of the putative ORF5 stem loop and further search for similar chimeric viruses. We also would like to highlight the potential of recombination by administering PRRSV MLV vaccines to the domestic pig population. 

## 2. Materials and Methods

### 2.1. Cells 

Porcine alveolar macrophages (PAMs) were isolated by bronchoalveolar lavage (BAL) from euthanized pigs that tested free of PRRSV RNA with an RT-qPCR. First, the lungs and trachea were removed without damaging the tissue, to avoid the presence of erythrocytes in the BAL fluid. Then, sterile phosphate-buffered saline (PBS) was poured through the trachea into the lungs. After gently massaging the organ, the BAL fluid was poured into a glass bottle and kept on ice until further processing. Next, the fluid was centrifuged, and the pellet was washed 3 × with PBS. Finally, the cell number was determined, and batches of 1 × 10^8^ cells were frozen in FCS (FCS, Corning, NY, USA) + 10% DMSO (Carl Roth, Austria) at −150 °C until further use. African green monkey cells (MARC-145) were obtained from the Friedrich-Loeffler-Institute in Germany.

### 2.2. Virus Isolation by Cell Culture

Serum samples were briefly centrifuged at 10,000 × *g* and 30 µL were utilized to infect 2 × 10^6^ PAMs and 5 × 10^5^ MARC-145 cells in 24-well plates. An aliquot of the PAMs from the corresponding donor animal was previously tested for high susceptibility toward PRRSV. Infection was assessed after 48 h using immunofluorescence. Supernatants were collected and stored at 4 °C until further use, and cells were fixed with 4% PFA (Carl Roth, Germany) for 20 min at 4 °C. After permeabilization of cell membranes with 1% Triton-X 100 (Carl Roth, Karlsruhe, Germany) for 5 min at RT, cells were inoculated with an in-house-produced mAb anti-PRRSV N (clone P10/b1) for 1 h. A Cy3-linked (Dianova, Hamburg, Germany) detection serum was added to visualize PRRSV-infected cells. Cell supernatants were used for further virus passaging and confirmation of the immunofluorescence results by RT-PCR.

### 2.3. RT-PCR

Organ samples were homogenized in sterile PBS for 3 min at 30 oscillations/sec with a Tissue Lyser II (Qiagen, Hilden, Germany) and stainless-steel beads. Samples were centrifuged for 3 min at 13,000 rpm. Organ supernatants and serum samples were used for extracting nucleic acids with a QIAamp Viral RNA Mini QIAcube Kit in a QIAcube (Qiagen, Hilden, Germany) according to the manufacturer’s instructions. Extracts were used to detect PRRSV with an ORF7-specific RT-PCR as previously described [20].

### 2.4. Virus Propagation and RNA Extraction

Supernatants of infected cells (described in 2.2.) were used to infect more PAMs to produce a sufficient amount of virus for RNA extraction. Virus of the second passage on 5 × 10^7^ cells provided a total of 10^7^ TCID_50_ with titers of 2 × 10^5^/mL. 50 mL of each preparation was cleared by centrifugation at 10,000× *g* with a Fibrelite 14.2 rotor for 5 min and filtration through a 0.45 µm sterile filter (Pall, Port Washington, NY, USA). After that, the virus was concentrated in a Beckman Ti55.2 rotor (Beckman Coulter, Vienna, Austria) at 35,000 rpm for 2 h. Next, the pelleted material was resuspended overnight in 500 µL phosphate-buffered saline at 4 °C, and the insoluble debris was removed in a microcentrifuge at 10,000× *g* for 1 min. The supernatant was pelleted at 45,000 rpm in a TLA45 rotor at 4 °C for 1 h. The sediment was resuspended in 50 µL dH_2_O and lysed in 600 µL lysis solution of RNeasy kit (Qiagen, Hilden, Germany). All further steps were carried out according to the manufacturer’s instructions.

### 2.5. cDNA Preparation and Sequencing

Purified RNA samples were subjected to Sanger and Next Generation Sequencing (NGS). For Sanger sequencing, RNA was primed with oligonucleotide T20, and cDNA was produced with MuLV Reverse transcriptase (NEB, Ipswitch, MA, USA) at 42° for 2 h. The cDNA was purified with the NEB Monarch kit and used as a template for a series of 15 overlapping PCR fragments with primer pairs PRS328–345 (Appendix A, Table A1). PCR fragments were submitted to Sanger sequencing (Eurofins) using primers from either end. Using this technique, sequences were assembled to about 98%. For NGS, 1 µg of RNA was sent on dry ice to Clontech for 75-nucleotide (nt) paired-end Illumina sequencing with 2 × 10^6^ reads. NGS data were aligned to the preliminary Sanger sequence to yield a composite sequence that immediately showed all ORFs. The 5’ends were determined by 5’ RACEas reported earlier [20]. The Basic Local Alignment Search Tool (BLAST) of the National Library of Medicine (NIH) and CLC Genomics Workbench 22 (Qiagen, Hilden, Germany) were used for sequence alignments. 

### 2.6. Recombination Analysis and RNA Structure Predictions

The recombination analysis was performed with SimPlot version 3.5.1. [21] and RDP5 version 5.29 [22]. For similarity and recombination analysis, sequence alignments were performed using CLC Genomics Workbench 22 (Qiagen, Hilden, Germany), with a gap open cost of 10.0 and a gap extension cost of 1.0. An alignment of the recombinant strains and a vaccine strain was imported into SimPlot to perform a Kimura 2-parameter test, with Ingelvac PRRSFLEX^®^ EU (GenBank: KT988004) as a query sequence, 200 bp steps, and a transition/transversion (Ts/Tv) ratio of 2.0. For recombination analysis, sequence alignments were imported to RDP5. A full exploratory recombination scan using the RBG, GENECoNV, Bootscan, MaxChi, Chimera, SiScan, and 3Seq programs was performed using default parameters. RNA secondary structure predictions were made with LocARNA version 1.9.1 linking Vienna RNA package 2.3.2 [23,24,25].

## 3. Results

### 3.1. Isolation and Sequencing of Chimeric PRRSV Strains

During clinical outbreaks or routine surveillance of pig herds in Germany and Austria, three PRRSV isolates were collected that exhibited a suspiciously high homology to the Ingelvac PRRSFLEX^®^ EU upon ORF5 sequencing. The acronyms of the viral strains indicate the nation, year of isolation, and entry number in our files. To exclude the possibility of assessing a mixed virus population, PRRSV was isolated from the clinical samples on PAMs. In parallel, MARC-145 cells were inoculated with the clinical samples. GER18-258, AUT20-1664, and AUT22-97 could be isolated from PAMS showing a cytopathic effect and a positive immunofluorescence signal upon staining with a mAb anti-PRRSV N. None of the samples led to a productive infection of MARC-145 cells, indicating the absence of attenuated MLV vaccine or PRRSV-2 strains. Only a small number of PRRSV-1 strains replicate in monkey cells without prior adaptation. So far, we isolated only one virus showing this phenotype [26]. 

The determination of the genomic sequences was achieved by Sanger sequencing of PCR products and a 5’-RACE. Isolate GER18-258 harbors 15,088 nt, isolate AUT20-1664 15,030 nt, and isolate AUT22-97 15,073 nt in total. A BLAST analysis of the full genome sequences revealed an 89% homology of GER18-258 to isolate GER09-613 (KT344816.1), 88% homology of AUT20-1664 to Lelystad virus (NC_043487.1), and 93% homology of AUT22-97 to isolate AUT15-33 (MT000052.1). Together with the immunofluorescence results, this confirmed that all three isolates are Betaarterivirus suid 1, or PRRSV-1, strains. To assess the origin and potential pathogenicity of these isolates, veterinarians handling the farms were contacted and a clinical report was compiled.

### 3.2. Anamnesis and Clinical Findings in the Affected Farms

**Figure 1:**
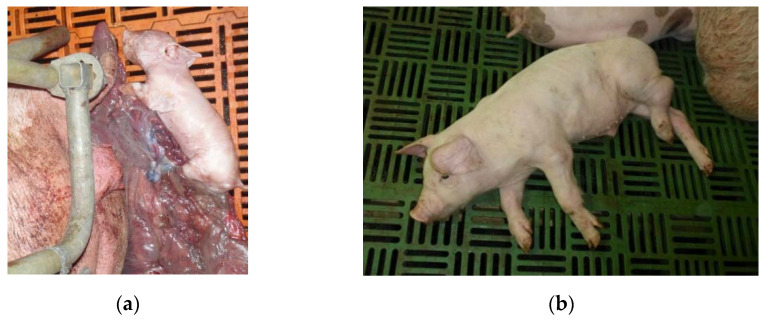
PRRS affected pigs on the farm of isolate GER18-258. (**a**) Stillborn piglet (**b**) Pig with swollen joints.

GER18-258 was derived from a farrow-to-finish farm in Southern Germany. The sow and nursery units were known to be free of PRRSV for more than ten years, while no PRRSV vaccines were applied. Reproductive disorders characterized by stillborn piglets (5%, Figure 1a) and weak born piglets (20 %) occurred. Approximately 30% of the sows in the farrowing unit were off feed and showed fever. In the affected batch, 70% of the suckling piglets died prior to weaning, and in the two consecutive farrowing batches the pre-weaning mortality was 50% and 30%. Based on clinical examination, approximately 30% of the nursery pigs and 40% of the fattening pigs exhibited the following symptoms: coughing, sneezing, increased respiratory rates, dyspnea, and conjunctivitis. In addition, swollen joints (Figure 1b) were noticed in individual nursery and fattening pigs. All-cause mortality increased from 3% to 5.5% in the nursery and from 2.5% to 5% in the fattening unit. PRRSV was detected in the lung and lymph nodes of six necropsied piglets with a commercial RT-PCR kit. Bacterial isolation from the lung tissue revealed growth of *Streptococcus suis* and *Staphylococcus aureus*. *Streptococcus suis* was also found in the swollen joints of the nursery pigs. 

AUT20-1664 was obtained from an Austrian nursery unit with pigs from two different sow farms. Sows from farm A were vaccinated against PRRSV (ReproCyc^®^ PRRS EU, Boehringer Ingelheim Vetmedica GmbH, Germany) every three months after the introduction of a new wild-type virus strain led to a severe PRRS outbreak in 2015. Piglets from this herd were vaccinated against PRRSV (Ingelvac PRRSFLEX^®^ EU, Boehringer Ingelheim Vetmedica GmbH, Germany) at three weeks of age. Piglets from farm B were vaccinated against PRRSV with the same vaccine when entering the nursery unit at the age of approximately 6.5 weeks. About 1% of the piglets exhibited respiratory symptoms and delayed growth. Diagnostic investigations of such runt pigs revealed mixed infections of PRRSV, PCV2 (no further genotyping done), and Influenza A virus (swine H1N1 of avian origin). During the bacteriologic investigation of lung samples, *Streptococcus suis*, *Pasteurella multocida,* and *Mycoplasma hyorhinis* could be isolated.

AUT22-97 was isolated in 2022 in an Austrian piglet-producing farm after facing respiratory distress and increased mortality in the nursery unit. Prior to PRRSV detection, the PRRSV status of the farm was unknown, and no PRRSV vaccine was administered. The clinical signs started in the rearing period with respiratory distress, wasting, and increased mortality rates of up to 10%. PRRSV antibodies of 10-week-old piglets were tested with positive results in 10/10 samples. A PRRSV-1 ORF1 RT-qPCR was performed in pools of five with positive results. Besides PRRSV, an infection with *Actinobacillus pleuropneumoniae* could be confirmed in the lung tissue of affected pigs. In addition, four sows aborted while clinical signs occurred in the nursery unit. 

More detailed clinical reports can be found in the Supplementary File S1.

### 3.3. PRRSV Isolates Are Recombinant Viruses Harboring ORF5–3′ Sequences from a Particular Modified Life Vaccine Strain 

**Figure 2:**
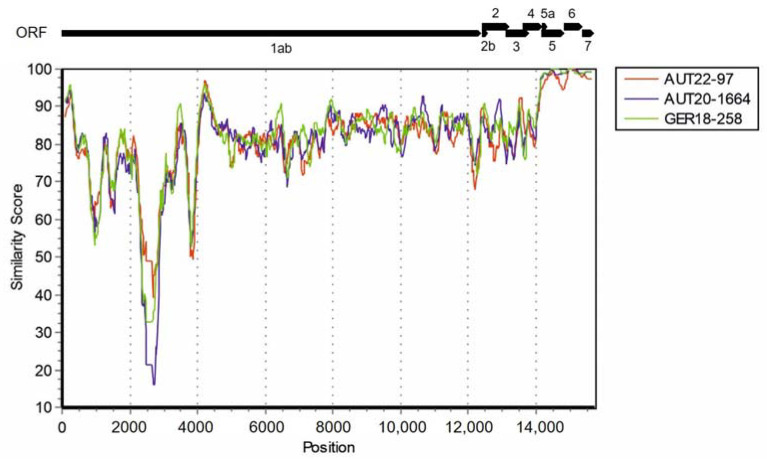
Similarity plot of three PRRSV isolates compared to MLV vaccine PRRSFLEX. A Kimura 2-parameter test with a transition/transversion (Ts/Tv) ratio of 2.0 was performed with the PRRSFLEX sequence as a query. The scheme above the plot shows the positions of the PRRSV open reading frames (ORFs).

To assess the genomic structure of the chimeric isolates, we conducted a full genome similarity analysis of the Ingelvac PRRSFLEX^®^ EU sequence to our recombinant isolates (Figure 2). This revealed a breakpoint of all isolates upstream of ORF5a. From this position onwards, the 3’-end matched the vaccine strain, while the 55’-end matched other PRRSV-1 strain sequences. Hence, we concluded that only a single crossover occurred.

**Figure 3:**
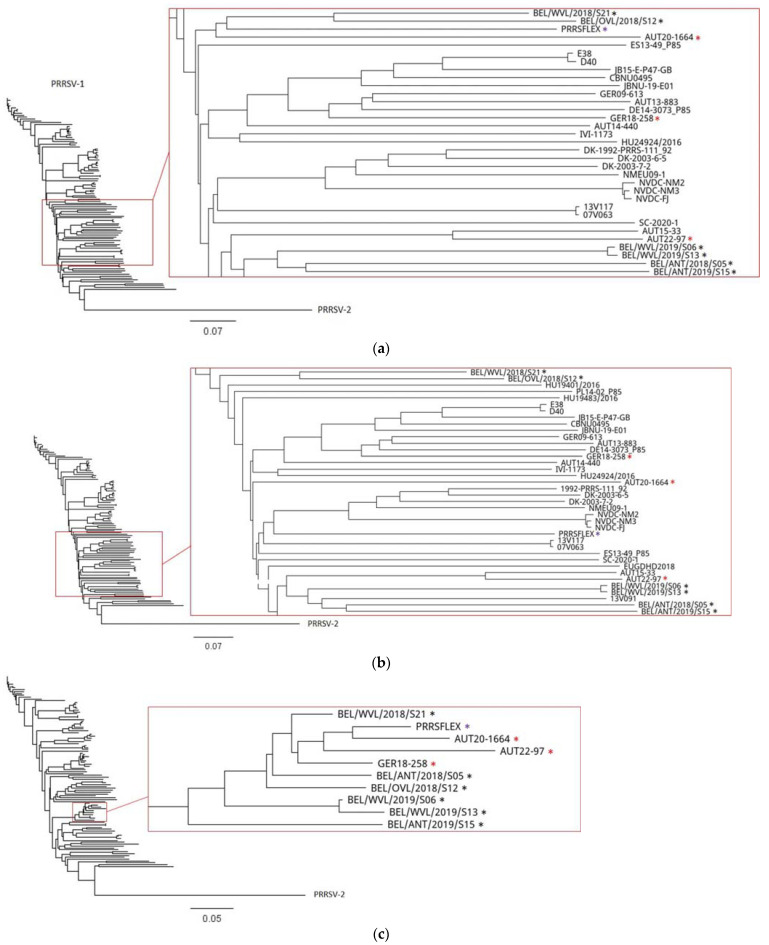
Phylogenetic trees of common PRRSV strains (Supplementary File S2) and our recombinant isolates (GER18-258, AUT20-1664, AUT22-97). The neighbor-joining method was used to generate trees of (**a**) whole genomes, (**b**) ORF1–4, and (**c**) ORF5–7. Individual PRRSV-2 strains are not shown, since our recombinants cluster is within PRRSV-1. Nucleotide distance was measured with the Jukes–Cantor model, and bootstrap analysis was performed with 100 replicates. Red asterisks mark our recombinant isolates, purple asterisk the Ingelvac PRRSFLEX^®^ EU strain, and black asterisks recombinant Ingelvac PRRSFLEX^®^ EU isolates described by Vandenbussche et al. [11].

A phylogenetic tree of a whole genome sequence alignment including the most common PRRSV-1, PRRSV-2, and PRRSV vaccine strains is shown in Figure 3a. A tree of the ORF1–4 (Figure 3b) disclosed only a clustering of isolate GER18-258 with the German isolates GER09-613 and DE14-3073_P85 and Austrian isolate AUT13-883. Isolate AUT20-1664, which showed the highest percent identity of only 88.3% with the Lelystad virus upon BLAST search, does not cluster with any given strain in our ORF1–4 phylogenetic tree. The ORF1-4 region of isolate AUT22-97 clustered with Austrian isolate AUT15-33. Repeating the same phylogenetic analysis with ORF5–7 (Figure 3c) revealed a clustering of all three isolates with the virus strain used for the Ingelvac PRRSFLEX^®^ EU vaccine (GenBank 94881) and six Belgian recombinant viruses described by Vandenbussche et al. [11]. These Belgian strains were identified as recombinant viruses between different wild-type strains and the Ingelvac PRRSFLEX^®^ EU vaccine strain with a recombination breakpoint within ORF5.

Next, we conducted a more thorough recombination analysis with the program RDP5, using six different recombination detection methods. This analysis revealed that GER18-258 is a derivative of strain GER09-613 as the major parent (88.9% similarity) and PRRSFLEX as the minor parent (99.4% similarity). The recombination breakpoint is predicted to be between nt 13,551 and 13,660. For isolate AUT20-1664, the analysis revealed an unknown major parent and PRRSFLEX as the minor parent (99.0% similarity), with a recombination breakpoint between nt 13,497 and 13,641. For AUT22-97, the program predicted AUT15-33 to be the major parent (92.5% similarity) and PRRSFLEX to be the minor parent (97.8% similarity), with the breakpoint between nt 13,510 and 13,616. Altogether, these findings let us conclude that isolate GER18-258 is most likely a recombinant of a distant parent of strain GER09-613 and the PRRSFLEX MLV vaccine, isolate AUT20-1664 a recombinant of a yet unassigned wild-type strain and the PRRSFLEX MLV vaccine, and AUT22-97 a recombinant between an AUT15-33 derivative and the PRRSFLEX MLV.

### 3.4. RNA Structure Predictions Reveal a Conserved Stem Loop Upstream of ORF5a

**Figure 4:**
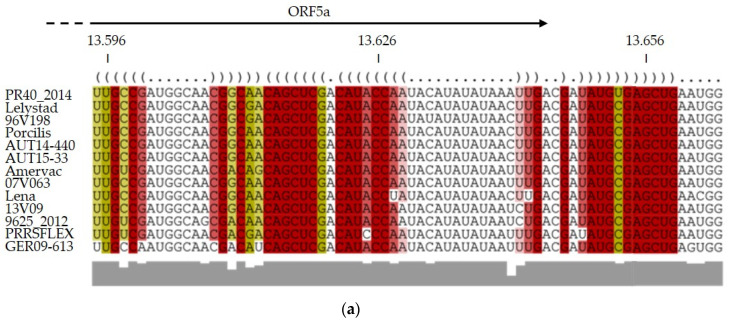

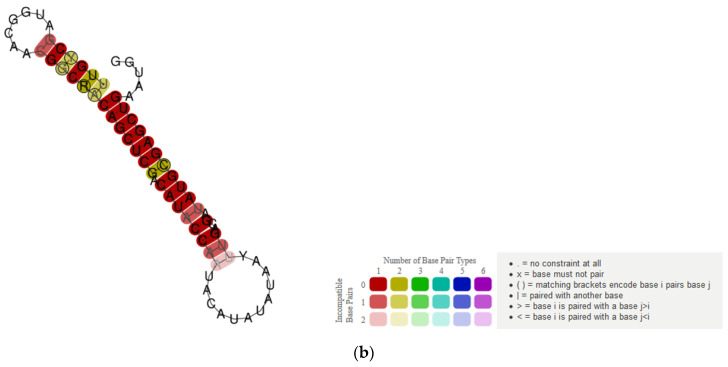
Predicted RNA folding of PRRSV sequence alignments. (**a**) Alignment and (**b**) predicted RNA folding of putative stem loop within ORF5 of several PRRSV-1 vaccines (Porcilis^®^ PRRS, Amervac^®^ PRRS, Ingelvac PRRSFLEX^®^ EU) and wild-type strains. Figures were created with LocARNA version 1.9.1.

The recombination analysis indicated that all three PRRSV isolates recombined within the same narrow genomic region with the same MLV vaccine strain. Therefore, we began to investigate the RNA secondary structure. Stem-loop structures are known to cause the RdRp to halt or become dislocated during replication [27]. We used the online tool LocARNA to upload an alignment of common PRRSV-1 vaccine and wild-type strains for RNA folding predictions (Figure 4a). The output revealed the presence of a stem loop upstream of ORF5a conserved in all given sequences (Figure 4b). Interestingly, this stem loop is conserved among a broad range of PRRSV-1, but not within PRRSV-2 strains. Since the minor parent of our three recombinant viruses, the PRRSFLEX MLV strain, also harbors this conserved stem loop, it is plausible that the RdRp has switched templates at this location in all three independent recombination events.

## 4. Discussion

A search utilizing keywords “PRRSV” [and] “recombination” yields more than 600 hits in PubMed^®^. Initially considered a rare event, recombination now appears to be a regular and, within the species *Betaarterivirus suid 1*, clinically relevant phenomenon. Attention was drawn to chimeric virus strains with the advent of severe clinical cases in China [28] and Denmark [19,29]. The latter case shook the field as two harmless vaccine strains had apparently recombined to create a virulent genotype. 

Here, we report on three different recombinant PRRSV-1 strains from geographically distant farms in Germany and Austria, isolated between 2018 and 2022. GER18-258 was identified in a farrow-to-finish farm in southern Germany after pigs exhibited clinical signs, such as respiratory distress, conjunctivitis, swollen joints, stillbirths, the birth of weak piglets, and increased pre-weaning mortality. AUT20-1664 was responsible for less severe clinical signs, mainly retarded growth and respiratory signs in piglets. AUT22-97 was detected in animals with respiratory distress, wasting, and increased mortality. In all three farms, co-infections with common respiratory bacteria or other viruses were detected. These often occur in PRRS outbreaks and can exacerbate the clinical outcome [30], as it was observed on the farm of isolate GER18-258 where *Streptococcus suis* caused swollen joints in nursery pigs. 

Initially, only part of the viral genomes was sequenced in all three cases. Routine detection and sequencing of PRRSV nucleic acid is usually achieved with an ORF5 or ORF7-specific RT-PCR. Considering isolate GER18-258, the initial sequencing of ORF5 led to the assumption that the vaccine strain was circulating in the herd. Only the full-genome sequencing revealed a possible crossover between a wild-type and a vaccine strain. This suggests that exclusive partial sequencing covering only one PRRSV ORF may lead to wrong assumptions regarding the present PRRSV isolate.

Recombination of PRRSV strains is facilitated by the ability of the RdRp to switch RNA templates during replication. One requirement for this copy-choice replication is that two (or more) strains infect the same host and the same cell. This scenario is not as far-fetched since it is not unusual that more than one PRRSV strain is present in a herd, and that PRRSV MLV vaccines are administered to infected pig herds as a metaphylactic measure. The Committee for Medicinal Products (CVMP) of the European Medicines Agency (EMA) outlined that the benefits of PRRSV MLV vaccines continue to outweigh their risks. The farm on which AUT20-1664 was isolated did indeed administer the Ingelvac PRRSFLEX^®^ EU vaccine prior to the isolation of the recombinant strain. Interestingly, on the farms where GER18-258 and AUT22-97 were isolated, no pigs were vaccinated with this particular product. Therefore, the option that the recombinant virus strains did not originate on these farms, but were introduced from another farm of unknown origin, has to be considered. This finding underlines the importance of accurate compliance with biosafety protocols on livestock farms. 

The recombination analysis confirmed our suspicion that the MLV Ingelvac PRRSFLEX^®^ EU is a recombination partner of all three isolates. While GER09-613 was determined as the major parent of isolate GER18-258, no major parent could be identified for isolate AUT20-1664, and AUT15-33 was predicted to be the major parent for isolate AUT22-97. For all three recombinants, the analysis revealed a recombination breakpoint at the beginning of ORF5 with Ingelvac PRRSFLEX^®^ EU as the minor parent. Despite the MLV vaccine part, the isolates retained their wild-type character and could be passaged on PAMs, but not on MARC-145 cells. Vandenbussche et al. [11] discovered similar PRRSV strains in 2021. The authors isolated 124 Belgian PRRSV-1 strains from pig sera to perform whole-genome sequencing. Eleven of those turned out to be vaccine virus recombinants, of which four displayed a recombination breakpoint with the Ingelvac PRRSFLEX^®^ EU strain within ORF5. These Belgian recombinant strains clustered with our recombinant isolates in the phylogenetic tree of Figure 3c. Since several PRRSV-1 isolates recombined at approximately the same location, we hypothesize that the RdRp did not randomly switch templates but used defined breakpoints or areas. 

However, we should not ignore the shaping power of evolution. Therefore, it is also necessary to ask what makes this genomic region so attractive for recombination and what evolutionary advantage the recombinants might carry. The PRRSV genome harbors a capped 5’-UTR with conserved RNA secondary structures, playing important roles in RNA replication and transcription [31,32,33]. Deletions in the 5’-UTR have resulted in reduced or absent replication and infectivity [34], leading to the conclusion that the stem loops within the 5’-UTR are involved in the RdRp-dependent replication process. Chang et al. [35] showed that the 5’ leader transcription regulatory sequence (TRS) of bovine coronavirus is located in the loop of a cloverleaf-like RNA structure, making it accessible for the RdRp. All *Arterivirus* 5’ leader TRSs are linked to the body TRSs in front of each individual ORF on the intermediate negative strand RNA [36]. The leader TRS base pairs with the body TRSs to initiate transcription of the subgenomic RNAs (sgRNAs). Subsequently, all polycistronic sgRNAs contain the 5’ leader sequence. These nested sgRNAs are templates for the structural proteins of the viral envelope, membrane protein, and nucleocapsid protein. RNA secondary structure predictions of several PRRSV-1 wild-type and vaccine strains let us hypothesize that ORF5 contains a conserved stem loop upstream of our isolates’ predicted recombination breakpoints. This loop could be the driving force for the RdRp to switch templates and produce a chimeric genome. While the stem-loop structure is a plausible localization for recombination events, it is most likely not its main purpose, since conserved stem-loop sequences often have regulatory functions [27]. Nevertheless, this hypothesis has to be explored by investigating stem-loop structure and function in more detail.

The question whether the recombination between field and vaccine strains occurs by chance or has a selective advantage remains unsolved. The most recent isolate, AUT22-97, derives from AUT15-33 that emerged seven years ago as an outbreak with severe reproductive losses and clinical signs in nursery pigs on Austrian farms [37]. AUT15-33 is characterized extensively and shows both considerable virulence in reproductive as well as respiratory challenge trials [20,38]. We frequently find descendants of AUT15-33 in current clinical samples from Austria indicating high competitiveness in the field. Hence, it is surprising that an already vital wild-type strain is outcompeted in the field by a recombinant equipped with MLV PRRSFLEX^®^ EU ORF5–7 sequences. The high number of altogether seven cases, including the Belgian chimeric viruses, indicates that there is something special to the recombined elements of Ingelvac PRRSFLEX^®^ EU. A simple immunological escape is unlikely as the immune response towards the vaccine strain, at least regarding ORF5–ORF7, is mostly unaffected. Future experiments, including reverse genetics of cloned AUT22-97 and AUT15-33 and infection trials to compare the clinical outcome of wild-type and chimeric viruses, will address this important question.

## 5. Conclusions

We report on three recombinant PRRSV strains isolated from geographically distant farms suffering from mild to severe clinical cases of reproductive or respiratory disorders. NGS and recombination analyses confirmed the parental strains of isolate GER18-258 to be GER09-613 from ORF1–4 and the MLV Ingelvac PRRSFLEX^®^ EU from ORF5–7. Isolate AUT20-1664 is a chimeric virus of an unknown strain providing ORF1–4 and the MLV Ingelvac PRRSFLEX^®^ EU contributing to ORF5-7. Strain AUT22-97 is a recombinant virus with AUT15-33 from ORF1–4 as the major and the MLV Ingelvac PRRSFLEX^®^ EU from ORF5–7 as the minor parent. RNA structure predictions of the sequences around the recombination breakpoints revealed a conserved stem loop upstream of ORF5a. We hypothesize that this stem loop might be the driving force of the RdRp template switch, resulting in the crossover events. It is unclear if the recombinants obtain an evolutional advantage compared to their parental wild-type strains from acquiring the MLV sequences. Further research on the pathogenicity and infectivity of the isolates and the molecular function of the putative stem loop has to be done.

## Data Availability

Viral sequences were submitted to GenBank^®^; submission numbers: OP627116, OP627117, OP970824.

## Supplementary Materials

The following supporting information can be downloaded at: https://www.mdpi.com/article/10.3390/v15010258/s1, Supplementary File S1: Anamnesis and physical findings [39]; Supplementary File S2: PRRSV accession numbers used for phylogenetic trees in Figure S3.

## Appendix A

**Table A1:** Primers used for Sanger sequencing of PRRSV isolates.

Primer	Sequence	Forward/Reverse	Binding at nt
PRS328	GYGAWCTYCAAGTTTAYGAGC	forward	527–547
PRS329	GAGCTCCAARCAGGCCATG	forward	2040–2059
PRS330	CCRATACTCGGATGCCTTCC	forward	3506–3527
PRS331	CCACGTGGTGCRGCTGCTG	forward	4993–5011
PRS332	AYGTRATYGTTCTGCTTGGGC	forward	6533–6553
PRS333	TGCCTGGGGTCCTACGCCT	forward	7984–8002
PRS334	GAYGATGATGTYATYTACACACC	forward	9636–9658
PRS335	CTCTCACCGATGTGTACCTY	forward	11,002–11,021
PRS336	CGTCCGGGTACGAYAAYCTY	forward	12,502–12,521
PRS337	GTGTCWCGCGGCCGACTCYTG	forward	14,004–14,024
PRS338	TGGTCRGACACGTGCATGGAG	reverse	606–626
PRS339	RGGCCTTKGAGGAKGGRAG	reverse	2081–2098
PRS340	GCCATCCAAGAACCAAAAACAC	reverse	3572–3593
PRS341	CTGAARGCACCTTCRAGRAGGG	reverse	5105–5126
PRS342	CATTRATRTCGAGGATGGATCC	reverse	6565–6586
PRS343	TGGCCATTRAYCCCTGCCA	reverse	8083–8102
PRS344	GGAATACCTRCAAACTTTRAGAGC	reverse	9690–9713
PRS345	TTCCAGCATTTTGAYGCCGTC	reverse	11,051–11,071
PRS346	MGGATGGAAYTGGGCCGCT	reverse	12,569–12,588
PRS347	AAATGCACATATGTCATGTAYCC	reverse	14,070–14,092
