# Supplementary material for: A Conserved Stem-Loop Structure within ORF5 Is a Frequent Recombination Hotspot for Porcine Reproductive and Respiratory Syndrome Virus 1 (PRRSV-1) with a Particular Modified Live Virus (MLV) Strain
Source: Viruses. 2023 Jan 16;15(1):258. doi: 10.3390/v15010258 (PMC9867337; doi:10.3390/v15010258)
Supplement: Supplementary file 1 [file viruses-15-00258-s001.zip › S1_Anamnesis_and_clinicall_findings.pdf]

Supplementary File S1

Anamnesis and clinical findings associated with GER18-258 occurrence

GER18-258 was derived from a farrow to finish farm in Southern Germany harboring 80 sows. The farm produces in a three-week farrowing interval. Piglets weaned at the age of 28 days are nursed on site and transferred to the fattening unit with a body weight of 28kg. The fattening unit with 1500 places is located at a distance of 2km from the remaining buildings and managed by different personnel. In addition to the on-farm produced fattening pigs 150 fattening pigs are purchased every 3 weeks from different sources. The sows and gilts are vaccinated against Porcine Parvovirus and Erysipelas according to the manufacturer's recommendation. Vaccination protocol of piglets includes *Mycoplasma hyopneumoniae* and porcine Circovirus type 2 (PCV-2) in the third week of life. The sow and the nursery units are known to be free of PRRSV since more than ten years, based on routine serological testing of sows and nursery piglets performed every six months. In accordance with the detection of GER18-258 reproductive disorders characterized by stillborn piglets (5%, figure 1a) and weak born piglets (20 %) occurred. Approximately 30% of the sows in the farrowing unit were off feed and showed fever. In the affected batch, 70% of the suckling piglets died prior to weaning and in the two following farrowing batches the pre-weaning mortality accounted for 50% and 30%. Two weeks after the first occurrence of clinical signs in the farrowing unit respiratory distress was observed in the nursery and fattening unit. Based on clinical examination approximately 30% of the nursery pigs and 40% of the fattening pigs showed coughing, sneezing, increased respiratory rates, dyspnoea, and conjunctivitis. In addition, swollen joints (figure 1b) were noticed in individual nursery and fattening pigs. All-cause mortality in the nursery increased from 3% to 5.5% and from 2.5% to 5% in the fattening unit.

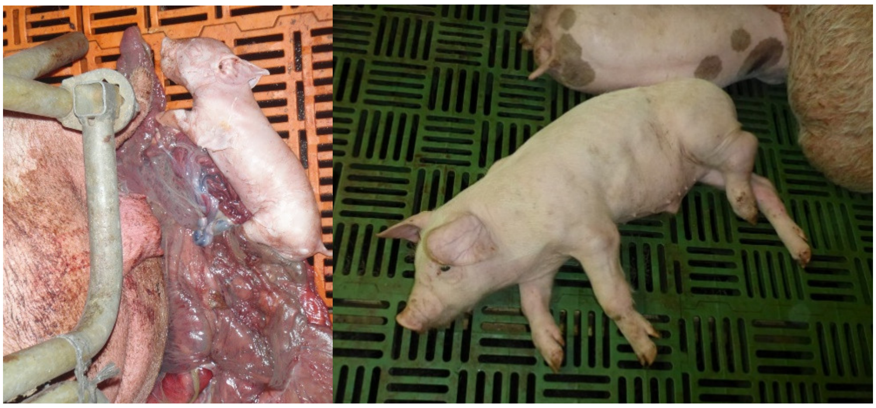

a) b)

Figure S1. PRRS affected pigs on the farm of isolate GER18-258. a) stillborn piglet, b) pig displaying swollen joints

For diagnostic workup blood samples from 10 sows, 10 pigs end of nursery and 10 fattening pigs were collected. Blood samples from sows were negative for antibodies against Influenza A and Leptospira. Antibodies against PRRSV were present in 8/10 sows, 8/10 nursery pigs and in all fattening pigs. Six weak born piglets were submitted for necropsy. PRRSV was detected in lung and lymph nodes samples of all six weak born piglets using a commercial RT-PCR kit. Post mortem examination of nursery and fattening pigs revealed poor retraction of the lungs in all animals as well as purulent arthritis in the nursery pigs. Histologically, interstitial pneumonia including hyperplasia of type II pneumocytes were found in the lungs of all investigated pigs. Bacterial isolation from lung tissue revealed growth of *Streptococcus suis* and *Staphylococcus aureus*. *Streptococcus suis* was also found in the joints of the nursery pigs. PRRSV was detected in lung and tonsil samples, whereas all investigated samples were negative for PCV-2 and IAV.

Initial phylogenetic analysis of two qRT-PCR PRRSV positive samples was performed at IVD GmbH, Seelze, Germany using ORF 5 sequencing. ORF 5 sequencing revealed 97% nucleotide identity to Ingelvac PRRSFLEX® EU (Boehringer Ingelheim Vetmedica GmbH, Germany) for sample one and 99% nucleotide identity to Ingelvac PRRSFLEX® EU for the second sample. The nucleotide identity between

these two samples and PRRSV-1 prototype strain Lelystad virus (LV) was only 91% and 95% respectively.

Anamnesis and clinical findings associated with AUT20-1664 occurrence

Isolate AUT20-1664 was obtained from a nursery unit, which was newly built in 2019; piglets from two different sow farms of the same owner are housed in this unit from seven weeks of age until 30 – 35 kg body weight ,when they are sold to different finishing sites. The two sow farms both produce in a four-week batch farrowing interval with a two-week period in between farrowings of the two farms. Sow farm A operates with 350 sows and is classified as PRRS stable according to Holtkamp *et al.* [1]; sows are vaccinated against PRRSV (ReproCyc® PRRS EU, Boehringer Ingelheim Vetmedica GmbH, Germany) every three months after the introduction of a new field virus strain led to a severe PRRS outbreak in 2015. Piglets from this herd are vaccinated against PRRSV (Ingelvac PRRSFLEX® EU, Boehringer Ingelheim Vetmedica GmbH, Germany) at weaning (three weeks of age), when they are transferred to a weaning unit located within the sow farm. After three and a half weeks, piglets are moved to the newly built nursery unit which is located about 300 meters from the sow farm. Sow farm B produces piglets with 600 sows and is free of PRRS based on routine serological testing; piglets from this herd are housed in a nursery unit within the sow farm for 3.5 weeks after weaning before they are transferred to the newly built nursery unit located around 100 km from the sow farm. Piglets from farm B are vaccinated against PRRSV (Ingelvac PRRSFLEX® EU, Boehringer Ingelheim Vetmedica GmbH, Germany), when entering the nursery unit at the age of approximately 6.5 weeks. The set-up led to the placement of piglets with different PRRS status, i.e. vaccinated 3.5 weeks prior to entry or vaccinated directly at entry, into the nursery unit every other week. The nursery unit consisted of four different rooms with 6 pens in each room to house a total of 2600 nursery piglets. No strict all-in/all-out was performed and piglets from the two sources shared the same air space. According to the farmer, production parameters in the nursery were satisfying with 1-2 % average piglet all-cause mortality. Nevertheless, about 1 % of the piglets showed respiratory symptoms and retarded growth. Diagnostic investigations of such runt pigs revealed a mixed infection with PRRSV, PCV2 (no further genotyping done) and Influenza A virus (swine H1N1 of avian origin). Histologic lesions of the lung were described as purulent bronchopneumonia, peribronchial interstitial pneumonia and partially severe damage of alveoli with type 2 pneumocyte proliferation. Pathologists summarized their report as lung lesions caused by viral infection with secondary bacterial colonization. Following bacteriological examination *Streptococcus suis*, *Pasteurella multocida* and *Mycoplasma hyorhinis* could be isolated.

Anamnesis and clinical findings associated with AUT22-97 occurrence

In January 2022, an Austrian piglet-producing farm harboring 70 sows faced respiratory distress and increased mortality in the nursery unit. The farm is located in Styria, the southern part of Austria, in an area with a high density of pig farms. The farm has a three-week batch farrowing rhythm and a suckling period of 28 days. Approximately 26 piglets are weaned per sow and year. Gilts are bought from a conventional gilt-producing farm with unknown PRRS-status. After arrival on the farm, all gilts are kept in isolation units for six weeks. During isolation, gilts are not routinely tested for the presence of certain pathogens or antibodies against common pathogens, like PRRSV. Semen is acquired from a conventional boar stud located in Styria; one teaser boar is kept in the service center. Cleaning and disinfection are performed between batches in the farrowing rooms as well as in the nursery, whereas a strict "all-in/all-out" is not possible in the nursery unit, since two age groups have to be kept together for structural reasons.

The sows and gilts are vaccinated against porcine parvovirus and Erysipelas (Parvoruvac®, Ceva Santé Animale, France) according to the manufacturer's recommendation. Vaccination protocol of piglets includes *Mycoplasma hyopneumoniae* (Hyogen®, Ceva Santé Animale, France) and PCV-2 (Ingelvac CircoFLEX®, Boehringer Ingelheim Vetmedica GmbH, Germany) in the third week of life. Additionally, an inactivated vaccination against *Escherichia coli* and *Clostridium perfringens* (Enteroporc Coli AC, Ceva Santé Animale, France) is used in sows for passive immunization of piglets. Prior to the PRRSV detection, the PRRSV-status of the farm was unknown. Investigated thymus pool samples from

aborted fetuses were PRRSV negative by PCR in 2018. The farm has recurring problems with post-weaning diarrhea. In January 2022 hemolytic *Escherichia coli* (virulence genes for fimH-fimbriae, F4-fimbriae, heat-labile enterotoxin LT, and hlyA-hemolysin) were detected. Colistin was used for treatment.

The clinical signs started in the rearing period. Respiratory distress, wasting and increased mortality rates up to 10% were observed. The herd-attending veterinarian suspected an outbreak of *Actinobacillus pleuropneumoniae*, which could be confirmed in necropsy and sampling of lung tissue. Additionally, 10 serum samples from 10-week-old nursery pigs were taken and investigated for PRRSV as predisposing pathogen. Antibodies were investigated by IDEXX PRRS X3® ELISA (IDEXX PRRS X3® Ab Test, IDEXX Europe B.V., Netherlands), with positive results in 10/10 samples (S/P ratios ranged from 1.56 to 2.25; cut-off: 0.4). PRRSV1 ORF1 RT-qPCR was performed in pools of five with positive results (2.84×10^7 genome equivalents [GE]/mL and 4.85×10^7 GE/mL). In addition, four sows aborted at the same time clinical signs occurred in the nursery unit. Increased return-to-heat and increased numbers of stillborn or weak born piglets could not be observed. Fattening pigs didn't show respiratory signs, but tail biting could be observed.

Immediately after occurrence of clinical signs, all gilts and sows were vaccinated against *Actinobacillus pleuropneumoniae* (Coglapix®, Ceva Santé Animale, France) and against PRRSV (UNISTRAIN® PRRS, Laboratorios Hipra, S.A., Spain). Additionally, all piglets were routinely vaccinated against PRRSV (UNISTRAIN® PRRS, Laboratorios Hipra, S.A., Spain) within the third week of life. After mass vaccination, gilts are vaccinated twice against PRRSV in the isolation unit. The sows are re-vaccinated every four months. Clinical signs in the nursery vanished after three batches and production parameters reached levels prior to the outbreak. The next batch of gilts was sampled within quarantine (April 2022), with negative PRRSV antibody and PCR results.

References

1. Holtkamp, D.J.; Yeske, P.E.; Polson, D.D.; Melody, J.L.; Philips, R.C. A Prospective Study Evaluating Duration of Swine Breeding Herd PRRS Virus-Free Status and Its Relationship with Measured Risk. *Prev. Vet. Med.* **2010**, *96*, 186–193, doi:10.1016/j.prevetmed.2010.06.016.
